# Clinical trials and drug cost savings for Italian health service

**DOI:** 10.1186/s12913-020-05928-6

**Published:** 2020-11-26

**Authors:** Francesca D’Ambrosio, Gianfranco De Feo, Gerardo Botti, Arturo Capasso, Sandro Pignata, Piera Maiolino, Maria Triassi, Antonio Nardone, Franco Perrone, Michela Piezzo, Antonio Maria Grimaldi, Ida Palazzo, Immacolata De Stasio, Roberta D’Aniello, Alessandro Morabito, Giacomo Pascarella

**Affiliations:** 1 Scientific Directorate, Istituto Nazionale Tumori “Fondazione G. Pascale”, IRCCS, Naples, Italy; 2 School of Banking Wyższa Szkoła Bankowa, WSB University in Wrocław, Wrocław, Poland; 3 Department of Urogynecology, Istituto Nazionale Tumori “Fondazione G. Pascale”, IRCCS, Naples, Italy; 4 Hospital Pharmacy, Istituto Nazionale Tumori “Fondazione G. Pascale”, IRCCS, Naples, Italy; 5 Department of Public Health, Università Federico II, Naples, Italy; 6 Clinical Trials Unit, Istituto Nazionale Tumori “Fondazione G. Pascale”, IRCCS, Naples, Italy; 7 Breast Cancer, Istituto Nazionale Tumori “Fondazione G. Pascale”, IRCCS, Naples, Italy; 8 Melanoma, Cancer Immunotherapy and Innovative Therapies Unit, Istituto Nazionale Tumori, “Fondazione G.Pascale” – IRCCS, Naples, Italy; 9 Thoracic Medical Oncology, Istituto Nazionale Tumori, “Fondazione G.Pascale”, IRCCS, Via Mariano Semmola, 52 –, 80131 Naples, Italy

**Keywords:** Clinical trials, Cost savings, Treatment costs, Investigational drug, Per-patient cost, Drugs expenditure, Cost reduction

## Abstract

**Background:**

The cost of anticancer drugs is constantly growing. The aim of this study was determine the impact in terms of cost reduction for anticancer drug in the Italian Health Service due to patient participation in clinical trials.

**Methods:**

We evaluated the cost of drugs administered to patients treated in clinical trials at the National Cancer Institute of Naples in a four-week time period. Patients with a diagnosis of different cancers were considered, including adjuvant therapy and treatment for advanced disease, pharma sponsored and investigator initiated phase I, II and III clinical studies. We defined the expected standard treatment for each patient and we calculated the cost of the standard antineoplastic drugs that should be administered in clinical practice outside clinical trials. We used the market price of drugs to determine the cost savings value. Costs other than drugs were not included in the cost saving calculation.

**Results:**

From 23.10.2017 to 17.11.2017, 126 patients were treated in 34 pharma sponsored and investigator initiated clinical trials, using experimental drugs provided free of charge by the sponsors, for an overall number of 152 cycles of therapy. If these patients were treated with conventional therapies in clinical practice the cost of antineoplastic drugs would account for 517,658 Euros, with an average of 5487 Euros saved per patients for a period of 4 weeks.

**Conclusions:**

Clinical trials with investigational antineoplastic drugs provided free of charge by Sponsors render considerable cost savings, with a tangible benefit in clinical and administrative strategies to reduce drug expenditures.

## Background

The cost of anticancer drugs is constantly growing. The pharmaceutical expense for anticancer drugs has increased in Italy from 3.6 to 5 Billion Euros from 2013 to 2017, leaping to + 659 million Euros only in 2018. This growth ensures that all patients in Italy are able to access the best anticancer therapies. In 5 years (2013–2017) 54 new anti-cancer treatments were marketed worldwide and Italy has guaranteed (by 2018) the availability of 35 of these innovative molecules, ranking fifth internationally after the United States (52), Germany (43), United Kingdom (41), France (37), and ahead of Canada (33), Spain (30) and Japan (29) [1]. At a global level, this increase reached 133 Billion Dollars in the world in 2017 (versus 96 Billion Dollars in 2013). The conventional explanation among health economists is that the relentless rise in health care spending is driven by the development and diffusion of new drugs, devices, procedures, and ways of caring for patients [2]. Therefore, many hospitals are looking for ways to reduce costs and to avoid their spiraling expenses and survive by eliminating unnecessary discretionary and non-value adding costs [3]. For this purpose, health organizations can use two efficient tools, namely cost control and cost reduction. Cost control can be defined as the process of controlling how much a company or organization spends, so that costs are not greater than the agreed budget. It is a process of avoiding wasteful use of valuable resources and encouraging efficiency and cost consciousness, providing the necessary information to management concerned with keeping expenditures within acceptable limits [3]. Cost reduction, instead, is the process of reducing the amount of money that a company spends on wages, production, services, etc. in order to make it more profitable or a planned positive approach to reducing expenditures without compromising its quality [4]. The aim of this study was determine the impact in terms of drug cost reduction determined by patient participation in clinical trials, with investigational drugs provided free of charge by the Sponsors at an Italian Cancer Institute.

## Methods

### Patients and clinical trials

We evaluated patients treated with anti-neoplastic drugs within clinical trials at the National Cancer Institute of Naples, in a period of 4 weeks. All the clinical trials ongoing at the National Cancer Institute of Naples were considered for this analysis. Clinical trials included patients with different cancers, treated in both adjuvant and metastatic settings, in different phases of clinical research (phase I, II and III clinical trials). Both pharma sponsored and investigator initiated clinical studies were considered for this analysis: for pharma sponsored clinical trials, all drugs were provided at no cost by the relevant sponsors, while the experimental drug administered to patients enrolled in investigator-initiated clinical trials was provided free of charge by the marketing authorisation holder.

### Assessments

Drug dosage (in milligrams), number of effective administrations in the four-week period for each patient (cycles number) and market price of drugs were provided by the Hospital Pharmacy, including value added tax (VAT), as cost per milligram in the year of analysis. The market price was the actual price charged to the Hospital. The expected standard treatment was defined through interviews with the principal investigators who identified the standard antineoplastic drugs that should be administered in clinical practice outside clinical trials according to AIOM (Italian Association of Medical Oncology -Associazione Italiana Oncologia Medica) guidelines. The estimated cost for expected standard treatment was calculated considering the number of standard drug administrations foreseen by the standard schedule of treatment in a 4 week period.

By using the market price, we calculated the “per patient experimental drug cost” by summing the cost of each experimental drug administered per patient (Additional file 1).

### Analyses

Each experimental drug cost was obtained multiplying the numbers cycle * experimental drug patient dosage (mg) received * by market price experimental drug per mg. Adding up all “per patient experimental drug costs”, experimental drugs total cost was determined for all patients included in clinical trials. Then for each patient, we defined the expected standard treatment and we calculated the cost of the standard antineoplastic drugs that should be administered in clinical practice outside clinical trials. The “per patient standard treatment cost” was determined, multiplying the number of administered each standard drug treatment in absolute value * standard drug patient dosage per mg * by market price standard drug per mg (Additional file 2). For some patients, there was not a standard treatment and, therefore, the cost of conventional antineoplastic drugs was zero. The net cost savings over the duration of study period was calculated by adding the “per patient standard treatment cost” of both pharma sponsored and investigator initiated studies for all patients. The average per-patient cost savings for pharma sponsored and investigator initiated studies was defined by dividing “net cost savings” by the number of patients potentially candidates to standard therapy. We excluded patients not suitable of any conventional treatment from this calculation, because they would not have generated any cost for standard anticancer drugs. Costs other than drugs were (such as diagnostic tests, medical supplies, equipment and staff) not included in the cost savings calculation.

## Results

**Fig. 1:**
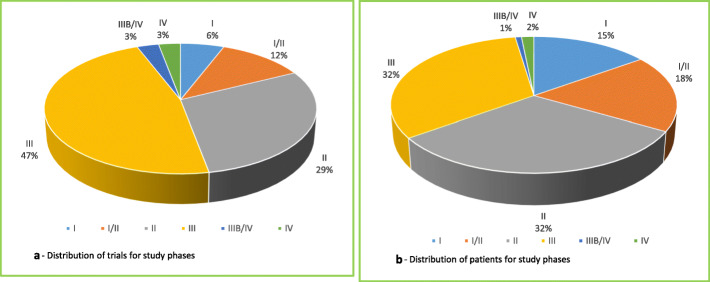
Distribution of trials (**a**) and patients (**b**) for study phases

**Fig. 2:**
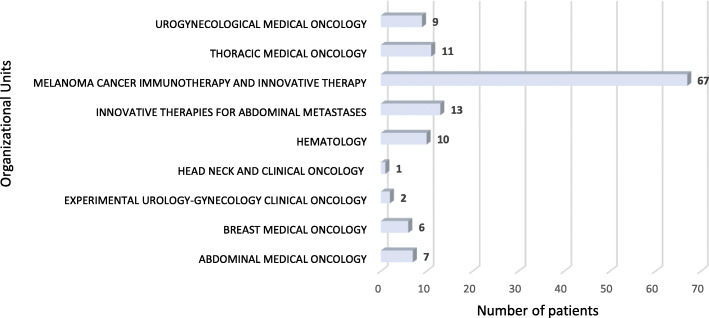
Clinical trials patients distribution for organizational units

**Fig. 3:**
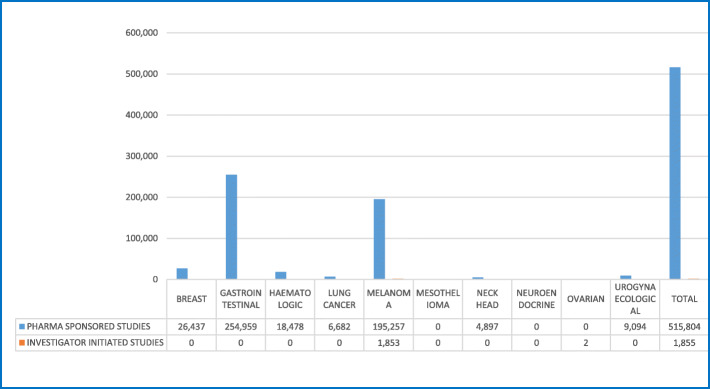
Drugs cost saving for cancer type

**Fig. 4:**
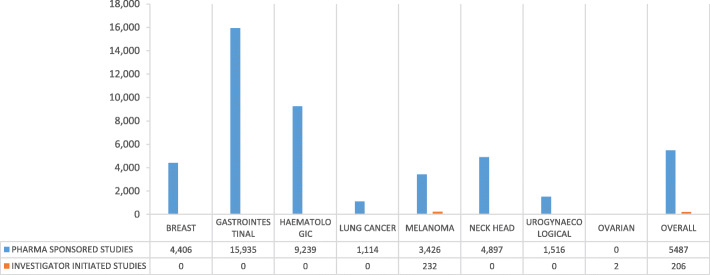
Per-patient average cost saving

**Fig. 5:**
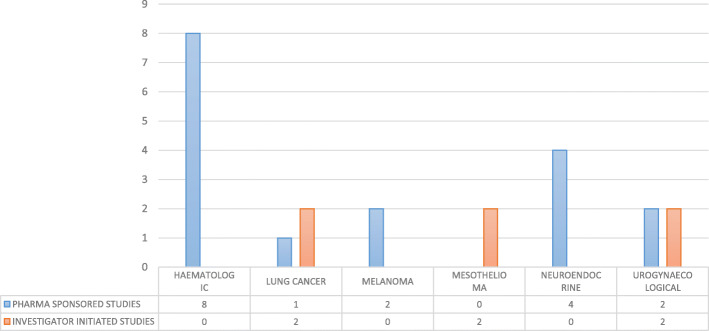
Patients with no standard therapy treated with experimental drugs

From 23.10.2017 to 17.11.2017, 126 patients were treated within 34 clinical trials for an overall number of 152 cycles of therapy at National Cancer Institute of Naples (Additional file 3). Phase III, II and I/II clinical trials were 47, 29 and 12%, respectively (Fig. 1a), while IIIB/IV and IV each only 3%. About 65% of patients were enrolled in Phase III and II clinical trials (Fig. 1b). Investigator initiated clinical trials were 15%, with 15 patients (12% of the total). Most of the patients (53%) were enrolled by the Unit for Melanoma Cancer Immunotherapy and Innovative Therapy (Fig. 2). Nivolumab was the experimental “drug” administered more frequently to patients (about 48%) and used in 10 clinical trials (29%), followed by pembrolizumab and atezolizumab used respectively in 7 (administered in 12,7% of patients) and 5 clinical trials (administered in 9,5% of patients). Seven of the experimental drugs used in clinical trials were not commercialized. They were administered to 24 patients (19% of the total), mainly with lung (25%) and urogynecological (25%) cancers. All investigator initiated clinical studies, instead, used commercialized drugs. Finally, 23 patients were untreatable with conventional alternative anticancer treatments. They were enrolled in 11 clinical studies, 3 of which were investigator initiated studies. Among these patients, 8 were treated with experimental drugs not yet commercialized within pharma sponsored clinical studies. The cost of experimental drugs administered to all patients included in clinical trials was 431,025 Euros (Additional file 2), 344,990 Euros (80%) for patients enrolled in profit clinical trials and 86,035 Euros (20%) for patients enrolled in investigator initiated clinical trials. The treatment of the same patients in the same 4-week period would have costed 517,658 Euros (Additional file 4) with conventional therapies outside clinical trials, 515,804 Euros (99,64%) with pharma sponsored clinical trials and 1855 Euros (0,36%) with non-profit clinical trials (Table 1). These costs would be mainly attributed to patients with gastrointestinal tumors (49% for pharma sponsored studies) and melanoma (38% for pharma sponsored studies) (Fig. 3). On average, the hospital saved 5.487 Euros per patient treated in pharma sponsored studies and 206 Euros for those treated within investigator initiated studies (Fig. 4). Finally, 23 patients did not have standard alternative treatment and the cost of conventional antineoplastic drugs for these patients was zero: among these patients, 17 were enrolled in pharma sponsored studies (8 of which were treated with experimental drugs not on the market) and 6 in investigator initiated studies (Fig. 5).

**Table 1:** Cost of drugs for standard treatments

	**Drug Cost of Standard Treatments**	
**Phatology type**	**Profit study**	**No profit study**
Breast	26.437	0
Gastrointestinal	254.959	0
Haematologic	18.478	0
Lung cancer	6.682	0
Melanoma	195.257	1.853
Mesothelioma	0	0
Neck head	4.897	0
Neuroendocrine	0	0
Ovarian	0	2
Urogynaecological	9.094	0
**Subtotal**	**515.804**	**1.855**
**Total**	**517.658**

## Discussion

This study evaluated the effect of pharma sponsored and investigator initiated clinical trials on hospitals’ pharmaceutical expenditure. In a four-week period 126 patients treated in clinical trials were analyzed in order to quantify drugs cost reduction related to the participation in clinical trials at National Cancer Institute of Naples. Our analysis demonstrated that the hospital has saved about 517,658 Euros in a month for drugs that, otherwise, would have been loaded on the Italian National Healthcare Service. If calculated with the same number of patients and in a one-year period, this saving could potentially translate into a significant drug cost reduction that exceed 6 million Euros, representing roughly 20% of the total spending for cancer drugs. To date, very few studies have evaluated the potential economic impact of patient participation in clinical trials in terms of drug cost savings, in particular with new immunotherapeutic and target based agents. This is a very important issue for health organizations, considering that the cost of anticancer drugs is constantly growing. Our study showed that participating in clinical trials with investigational anticancer drugs provided free of charge by the Sponsor translates into considerable cost savings, and tangible benefits in both clinical and administrative strategies for reducing drug expenditures. These findings should encourage the participation in clinical trials of an increasing number of Oncology Units (even outside Cancer Centers and Academic Institutions) overcoming the concern about the expected increased costs sustained by health organizations for conduction of clinical trials. McDonagh et al. [5] examined the costs and savings resulting from two pharmacy-based investigational drug services for fiscal years 1996–97. They showed that there was a cost avoidance of 2.9 Million Dollars in drug costs, which was equivalent to 8% of the institutions’ annual drug budget for 1996–1997. LaFluer et al. [6], through a review of the study protocols and dispensing data for the investigational drug studies over 2 years, have demonstrated that clinical trials participation achieves considerable drug cost avoidance, according to the type of study and the disease category involved. Uecke et al. [7] quantified drug cost savings in hospitals related to clinical trials and analyzed 88 clinical trials in oncology including 29 researchers in 11 hospitals in Germany from 2002 through 2005 with the aim to examine the relationship between researchers and hospital administrators with respect to clinical trials. The results showed a drug cost savings potential of 5.1 million Euros and an actual cost savings of 1.5 million Euros. In a retrospective cost attribution analysis to quantitate the treatment costs associated with cancer clinical trial protocols conducted in a single UK institution during 2009 and 2010 period, Liniker et al. [8] determined an overall treatment cost savings of 388,719 Pounds in 2009 and 496,556 Pounds in 2010, largely attributable to pharmaceutical company provision of free drug supplies. Grossi et al. [9], evaluated the cost of drugs administered in clinical practice and in clinical trials for a single Italian Lung Cancer Unit and they demonstrated that participation in clinical trials offers substantial cost savings for the Italian NHS related to drug provided free of charge by sponsors. They quantified these savings in 243,154 Euros (about 30% of the overall cost of those antineoplastic drugs charged to hospital during 2010). Calvin-Lamas et al. [10], carried out an observational of prevalence study with retrospective collected data related to prostate cancer clinical trials during the study period (1996–2013), demonstrating a total cost avoidance of 696,002 Euros and an average cost avoidance per patient was 5118 Euros. Finally, Manes-Sevilla et al. [11], in a retrospective observational study of the drug cost avoidance during the study period (2014–2016), calculated a total cost avoidance of 957,246 Euros and an average cost avoidance per patient of 10,756 Euros related to the free drugs supplied by the sponsors. They included thirty-seven clinical trials, with a total of 89 breast cancer patients, in this study. Our study confirmed these findings showing a significant cost savings related to investigational drugs that are provided to the hospital free of charge by the sponsor also in the era of immunotherapy. Other strengths of our study are the inclusion of patients with different cancers, in different settings of therapy (adjuvant and metastatic disease), treated within pharma sponsored and investigator initiated phase I, II and III clinical studies: all this allows for greater generalizability of data.

However, conducting clinical trials determines additional costs that inevitably loads on the hospital, which could affect the real economic benefit of drug cost saving. This represents a limit of the analyses conducted in our study, because we calculated the drug cost savings without taking into account the costs involved in the conduction of clinical trials. In our previous research [12], we estimated that for patients included in clinical trials, the average “per patient” total costs accounted for 11.379 Euros, including overhead costs of clinical trials. Nonetheless, it should also be considered that sponsored clinical studies are repaid entirely through sponsors’ grant (generally related to performing the activities envisaged by the protocol). Another weakness of our study could be the limited period of time chosen for the analysis. However, a 4 week period was empirically considered long enough for the purpose of the study, due to the high number of patients treated within clinical trials at our Cancer Center.

## Conclusion

Cancer clinical trials may provide a range of benefits for pharmaceutical companies, researchers and patients, but also for health care organizations and health systems. Our research demonstrated that treating patients within clinical trials lead to significant financial gains also in the hospital administrators perspective, leading to cost savings of conventional standard treatments and reducing drug expenditures.

### Aknowledgement

The Authors are grateful to Dr. Maura Tracey De Bellis for English revision and to Dr. Alessandra Trocino, Librarian at IRCCS “G. Pascale” of Naples, Italy, for the bibliographic assistance.

## Supplementary Information

**Additional file 1.** Experimental treatment database related to clinical trials.

**Additional file 2.** Standard treatment database related to clinical trials.

**Additional file 3.** Synthetic experimental and conventional treatment database.

**Additional file 4.** Synthetic experimental and conventional treatment costs database.

## Data Availability

Data and materials analyzed are available from the corresponding author on reasonable request.
